# 肺腺癌自噬相关基因预后风险评分模型构建及验证

**DOI:** 10.3779/j.issn.1009-3419.2021.103.09

**Published:** 2021-08-20

**Authors:** 静 周, 心悦 王, 兆娜 李, 日成 蒋

**Affiliations:** 300060 天津，天津医科大学肿瘤医院肺部肿瘤内科，国家肿瘤临床医学研究中心; 天津市“肿瘤防治”重点实验室; 天津市恶性肿瘤临床医学研究中心 Department of Thoracic Oncology, Tianjin Medical University Cancer Institute and Hospital, National Clinical Research Center for Cancer; Key Laboratory of Cancer Prevention and Therapy, Tianjin; Tianjin's Clinical Research Center for Cancer, Tianjin 300060, China

**Keywords:** 自噬相关基因, 肺肿瘤, 预后模型, *Cox*回归模型, LASSO回归, Autophagy related genes, Lung neoplasms, Prognostic model, *Cox* regression model, LASSO regression

## Abstract

**背景与目的:**

自噬相关基因(autophagy related genes, ARGs)可调控溶酶体的降解过程从而诱导细胞发生自噬，参与多种癌症的发生发展，肿瘤组织中ARGs的表达情况在预测患者生存方面具有很大的前景。本研究基于ARGs构建了肺腺癌(lung adenocarcinoma, LUAD)预后风险评分模型。

**方法:**

通过GeneCards数据库获得5, 786个ARGs。从癌症基因组图谱(The Cancer Genome Atlas, TCGA)数据库收集了395个LUAD患者的基因表达谱及临床数据，提取所有ARGs的表达数据，利用R软件筛选差异表达的ARGs。对差异表达的ARGs进行生存分析，筛选有预后价值的ARGs并进行功能富集分析。利用套索(the least absolute shrinkage and selection operator, LASSO)回归和*Cox*回归模型构建ARGs的预后风险评分模型。绘制受试者工作特征曲线(receiver operating characteristic curve，ROC曲线)得到风险评分的最佳cut-off值，将患者分为高风险评分组和低风险评分组。计算ROC曲线下面积(area under curve, AUC)和绘制*Kaplan-Meier*生存曲线评估模型性能，并在外部数据集验证。最后利用单因素和多因素*Cox*回归分析评价模型是否具有独立预后价值，并分析其临床相关性。

**结果:**

通过生存分析初步筛选了52个与预后相关的ARGs，以此为基础，利用LASSO回归和*Cox*回归分析构建了由5个ARGs(ADAM12、*CAMP*、DKK1、STRIP2和TFAP2A)组成的LUAD预后风险评分模型。该模型中，低风险评分组患者的生存时间明显优于高风险评分组(*P* < 0.001)，且在训练集(AUCmax=0.78)和两个外部验证集(AUCmax=0.88)中均展现出良好的预测性能。风险评分在单因素和多因素*Cox*回归分析中与LUAD患者预后显著相关(*P* < 0.001)，提示风险评分可作为LUAD潜在的独立预后因素。临床特征相关性分析表明高风险评分与高T分期、高肿瘤分期和发生不良预后密切相关。

**结论:**

我们构建了一个由5个ARGs组成的LUAD风险评分模型，该模型可为预测LUAD患者预后提供参考，未来或可与恶性肿瘤(tumor node metastasis, TNM)分期联合应用于肺腺癌患者的预后预测。

## Abstract (en)

**Background and objective:**

Autophagy related genes (ARGs) regulate lysosomal degradation to induce autophagy, and are involved in the occurrence and development of a variety of cancers. The expression of ARGs in tumor tissues has a great prospect in predicting the survival of patients. The aim of this study was to construct a prognostic risk score model for lung adenocarcinoma (LUAD) based on ARGs.

**Methods:**

5, 786 ARGs were obtained from GeneCards database. Gene expression profiles and clinical data of 395 LUAD patients were collected from The Cancer Genome Atlas (TCGA) database. All ARGs expression data were extracted, and The ARGs differentially expressed were identified by R software. Survival analysis of differentially expressed ARGs was performed to screen for ARGs with prognostic value, and functional enrichment analysis was performed. The least absolute selection operator (LASSO) regression and *Cox* regression model were used to construct a prognostic risk scoring model for ARGs. The receiver operating characteristic (ROC) curve was drawn to obtain the optimal cut-off value of risk score. According to the cut-off value, the patients were divided into high-risk group and low-risk group. The area under curve (AUC) and the *Kaplan-Meier* survival curve was plotted to evaluate the model performance, which was verified in external data sets. Finally, univariate and multivariate *Cox* regression analysis was applied to evaluate the independent prognostic value of the model, and its clinical relevance was analyzed.

**Results:**

Survival analysis, Lasso regression and *Cox* regression analysis were used to construct a LUAD prognostic risk score model with five ARGs (ADAM12, CAMP, DKK1, STRIP2 and TFAP2A). The survival time of patients with low-risk score in this model was significantly better than that of patients with high-risk score (*P* < 0.001). The model showed good prediction performance for LUAD in both the training set (AUCmax=0.78) and two external validation sets (AUCmax=0.88). Risk score was significantly associated with the prognosis of LUAD patients in univariate and multivariate *Cox* regression analyses, suggested that risk score could be a potential independent prognostic factor for LUAD. Correlation analysis of clinical characteristic showed that high risk score was closely associated with high T stage, high tumor stage and poor prognosis.

**Conclusion:**

We constructed a LUAD risk score model consisting of five ARGs, which can provide a reference for predicting the prognosis of LUAD patients, and may be used in combination with tumor node metastasis (TNM) staging for prognosis prediction of LUAD patients in the future.

肺癌是全球范围内癌症相关死亡的主要原因，其5年生存率约为15%。非小细胞肺癌占所有肺癌的75%-80%。其中，肺腺癌是非吸烟者和女性中最常见的非小细胞肺癌亚型，其危险因素主要包括二手烟、污染和职业致癌物，并具有遗传易感性^[1]^。尽管肺癌在化疗和靶向治疗方面已取得了很大的进展，但大多数患者的总生存率仍较低。其主要原因之一是大多数患者诊断时已处于晚期阶段。目前临床上常用的预测肺腺癌预后的指标包括肿瘤大小、转移情况和突变负荷等，但肿瘤存在高度的异质性，即使肿瘤原发灶-淋巴结-转移(tumor-node-metastasis, TNM)分期相同的患者，其治疗效果和预后仍有很大差别，单纯依靠上述指标有时并不能够准确预测患者预后，特异性欠佳。因此，我们需要探索新的生物标志物，可以辅助上述常用的预测指标，可靠的评估肿瘤患者预后和生存情况，为肺腺癌个体化诊疗提供依据。

自噬是由一系列ARGs调控的多步骤的溶酶体降解过程，已被广泛证实可参与多种癌症的发生发展^[2]^。大量研究表明，自噬在肿瘤的发生和治疗中是一把双刃剑。一方面，自噬可在细胞癌变前降解受损的细胞器以维持细胞稳态而发挥抑癌作用; 另一方面，自噬可促进细胞代谢物质循环，满足细胞营养需求，故在肿瘤发生晚期，自噬可为肿瘤细胞的增殖和侵袭提供能量和营养，提高肿瘤细胞对放化疗的耐受^[3]^。已有许多研究^[4, 5]^表明，自噬与肺癌的发生发展密切相关，如自噬相关基因10(ATG10)的过表达与肺癌的不良预后有关。尽管自噬在肿瘤治疗中的作用仍有争议，但已有证据^[6, 7]^表明，自噬是LUAD放化疗过程中关键的调控因子。

近几年，基于多个基因所构建的风险模型已被广泛研究并用于预测各种肿瘤的预后，如结肠癌、乳腺癌、肝细胞癌等，在某些癌种中，其预后预测性能甚至优于组织病理学诊断和肿瘤分期^[8, 9]^。本研究利用生物信息学，基于TCGA数据库筛选对肺腺癌有预后价值的自噬相关基因，通过*LASSO*和*Cox*回归分析最终构建了由5个ARGs组成的肺腺癌的预后风险评分模型，评估模型的预测性能，确定模型的独立预后价值和临床相关性，为肺腺癌患者的个体化诊疗提供参考。

## 1 资料与方法

### 1.1 数据获取

在GeneCards数据库(https://www.genecards.org/)中检索“Autophagy”获得5, 128个自噬相关基因。于2021年1月31日从TCGA数据库(https://portal.gdc.cancer.gov/)下载了全部的肺腺癌的3级RNA测序(RNA-Seq)数据。同时，从UCSC Xena网站(https://xenabrowser.net/)收集了这些患者相应的临床信息[包括性别、年龄、肿瘤大小、有无淋巴结转移、肿瘤分期、表皮生长因子受体(epidermal growth factor receptor, *EGFR*)基因突变、*ALK*基因融合、*KRAS*基因突变、生存时间及生存状态]。排除临床信息不完整的样本后，共纳入395例LUAD样本和48例正常样本。从基因表达综合数据库(Gene Expression Omnibus database, GEO)下载数据集GSE31210和GSE72094作为基因验证集。本研究中，所有数据集均来自公共数据库，无需伦理批准。

### 1.2 ARGs的差异表达分析和功能富集分析

利用R-4.0.3软件中的“limma”包筛选LUAD与正常肺组织间差异表达的ARGs，利用“edgeR”包对表达谱数据标准化，差异基因的筛选标准为：发现错误率(false discovery rate, FDR) < 0.05和|log2(Fold Change)| > 2。使用“ggplot2”和“pheatmap”包绘制火山图和热图用于差异基因的可视化。通过生存分析确定差异表达ARGs与总生存期(overall survival, OS)的关系，设置阈值为*P* < 0.05，初步筛选对肺腺癌有预后价值的差异表达自噬基因。利用R软件的“clusterProfiler”和“enrichplot”等包对差异基因进行基因本体功能(Gene Ontology, GO)富集分析和京都基因与基因组百科全书通路(Kyoto Encyclopedia of Genes and Genomes pathway, KEGG)分析及可视化，以探讨有预后价值的差异表达ARGs的潜在分子机制。*P* < 0.05被认为差异有统计学意义。

### 1.3 构建预后风险模型

通过log2(x+1)转换对RNA-Seq数据进行标准化，将差异表达ARGs的标准化表达量与LUAD样本生存信息合并，去除无生存时间记录的患者后，共381例LUAD样本纳入*Cox*回归分析。对有预后价值的ARGs进行单因素*Cox*回归分析、*LASSO*回归和多因素*Cox*回归分析并绘制森林图。*LASSO*回归可减少基因之间的共线性影响，降低后续所构建模型基因的过度拟合。在R软件中加载“glmnet”包，对经单因素*Cox*分析所获得的预后相关ARGs进行*LASSO*回归分析，将回归系数不为0的基因再纳入多因素*Cox*分析，构建最终的预后风险评分模型。风险评分(RiskScore)计算公式为：RiskScore=基因表达量_1_×Coef_1_+基因表达量_2_×Coef_2_+…+基因表达量_n_×Coef_n_(Coef：基因在多因素*Cox*回归分析中的回归系数，n：与预后相关ARGs总数目)。根据公式计算每位患者的风险评分，利用R软件的“pROC”包绘制受试者工作特征(receiver operator characteristic, ROC)曲线计算约登指数(Youden index)，以确定风险评分的最佳截断(cut-off)值。以cut-off值为截断值将肺腺癌患者分为高风险评分和低风险评分组。

### 1.4 评价预后风险模型

加载R软件“survival”包，根据上述分组绘制高、低风险评分分布曲线、生存情况分布图及建模基因表达量热图。同时，利用R软件的“survminer”包绘制*Kaplan-Meier*生存曲线对两组的OS进行生存分析比较。应用“timeROC”包绘制时间依赖性ROC曲线，分别计算样本OS在1年、2年、3年、5年、7年的AUC，从而评估模型预测预后的能力，AUC越高代表模型性能越好。

### 1.5 模型独立预后及临床特征相关性分析

对风险评分进行单因素和多因素*Cox*回归分析，以确定模型是否具有独立预后价值。若风险评分与OS在单因素和多因素*Cox*分析中均呈现显著差异，则说明风险评分可作为独立危险因素。将通过单因素和多因素分析识别出的独立危险因素作为变量，加载“rms”包，绘制列线图，用以推测患者未来的生存率。同时，分析风险评分是否对不同临床特征患者的预后也具有预测性能。

### 1.6 验证预后风险模型

在GEO数据库选取GSE31210和GSE72094作为外部验证集，根据上述风险评分公式，计算验证集中每个样本的风险评分。同样地，利用ROC曲线，确定两个数据集各自风险评分的最佳cut-off值，进行高、低风险分组，应用*Kaplan-Meier*生存曲线对两组的OS进行比较，AUC评估模型预测预后的能力，以验证预后风险模型的预测性能。

## 2 结果

### 2.1 差异表达自噬基因的筛选及功能富集分析

从GeneCards数据库中共收集5, 786个自噬相关基因。从TCGA数据库共收集395个肺腺癌组织和48个正常组织的mRNA表达数据。提取5, 786个ARGs的mRNA表达数据，以FDR < 0.05和|log2(Fold Change)| > 2为阈值，在肺腺癌与正常肺组织中共筛选出361个差异表达的ARGs，包括275个上调基因和86个下调基因，差异基因可视化结果如图 1A、图 1B所示。对上述361个差异基因进行生存分析，筛选出52个与预后显著相关的ARGs(*P* < 0.05)。对52个有预后价值的差异表达ARGs进行GO功能及KEGG通路富集分析，GO功能富集结果显示，差异表达ARGs主要富集在调控染色体分离、调控受体介导的内吞、调控细胞周期和调控自噬等(*P* < 0.05，图 2A); KEGG信号通路主要富集在细胞周期、细胞因子受体相互作用、IL-17信号通路、自噬、HIF-1信号通路和p53信号通路等(*P* < 0.05，图 2B)。

**图 1:**
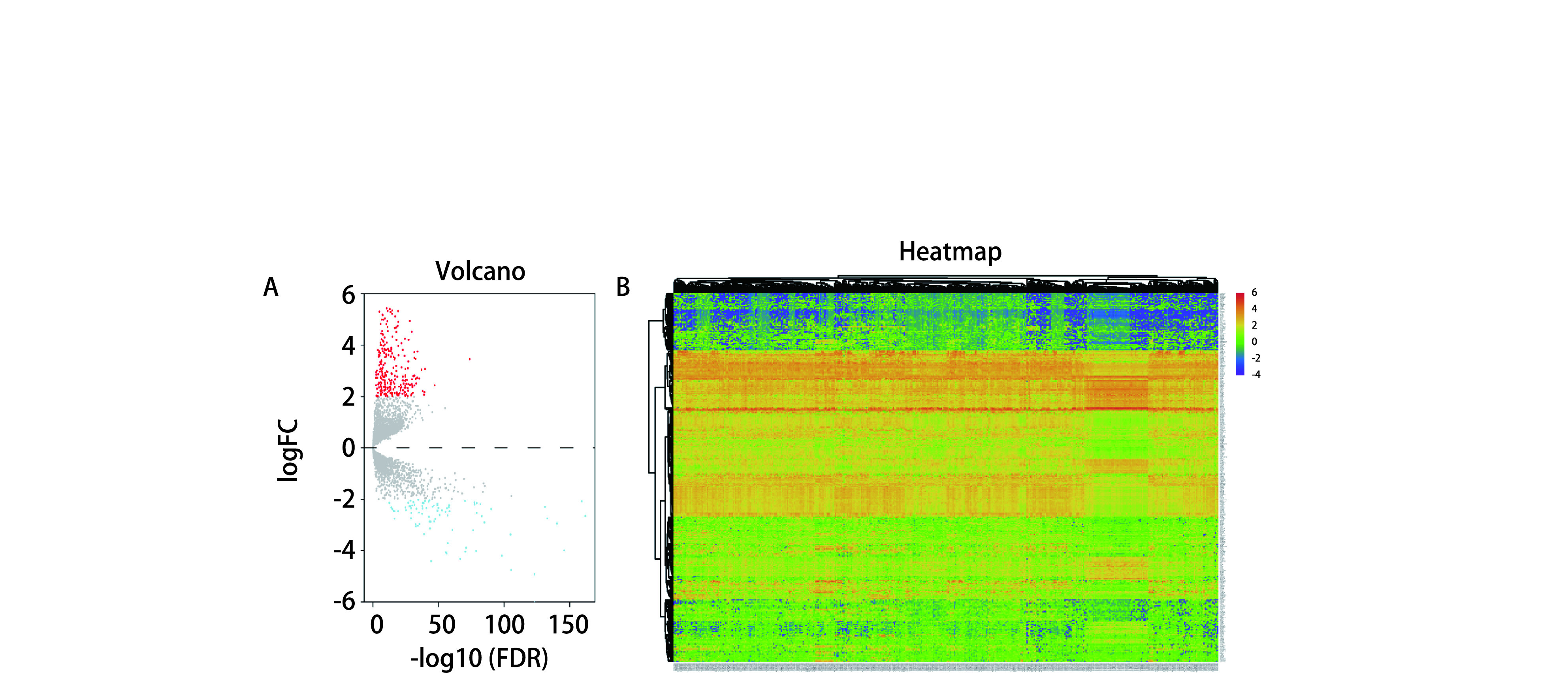
自噬相关基因的差异分析。A：差异表达基因火山图(275个上调基因和86个下调基因); B：差异表达基因表达热图。 Differential analysis of autophagy related genes. A: Volcano plot of differentially expressed genes (275 up-regulated genes and 86 down-regulated genes); B: Heat map of differentially expressed genes.

**图 2:**
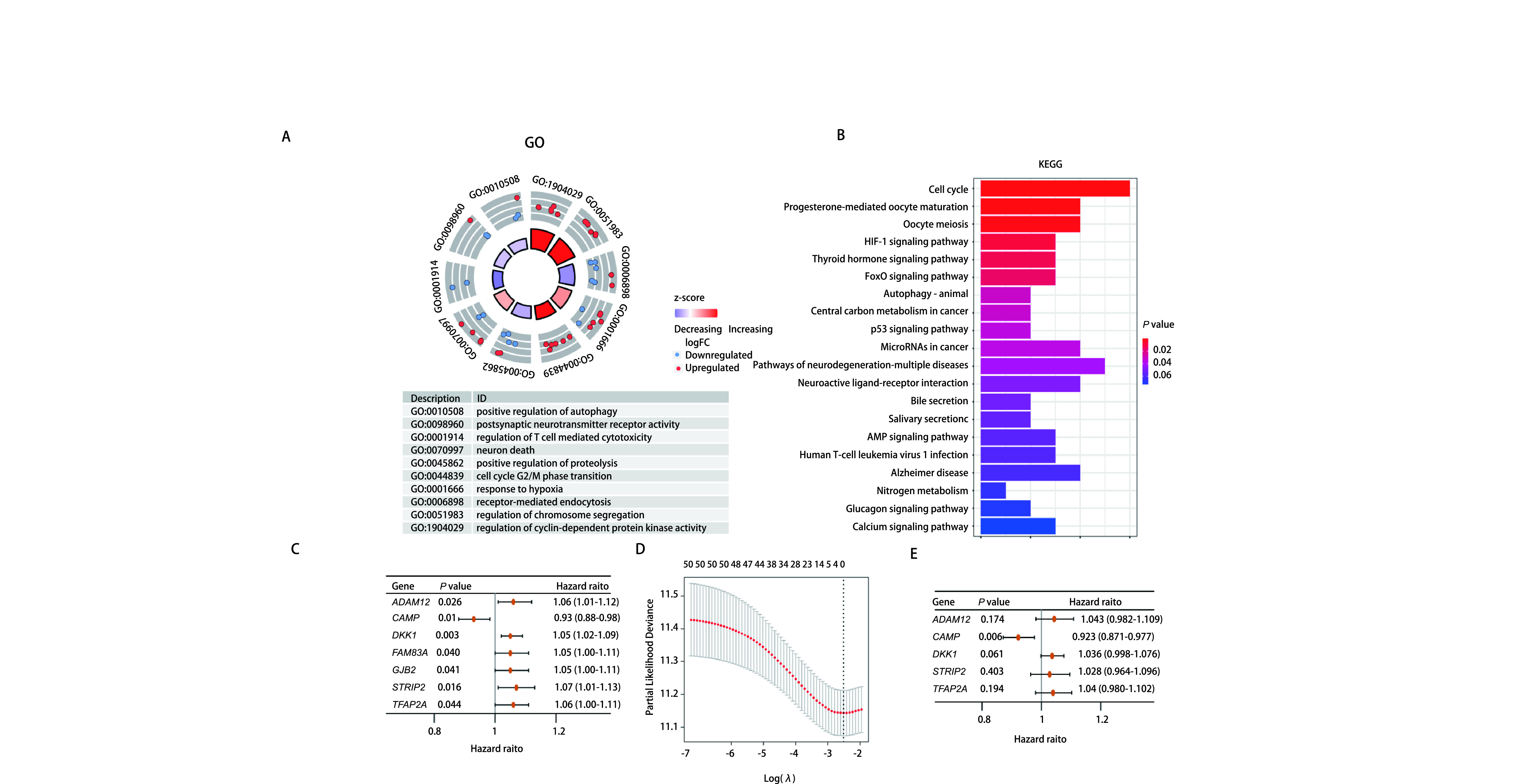
功能富集分析和预后风险评分模型的构建。A：GO富集分析; B：KEGG通路富集分析; C：单因素*Cox*回归分析结果森林图; D：LASSO分析; E：多因素*Cox*回归分析结果森林图。 Functional enrichment analysis and construction of prognostic risk score model. A: GO enrichment analysis; B: KEGG pathway enrichment analysis; C: The forest plots of univariate *Cox* regression analysis; D: LASSO analysis; E: The forest plot of multivariate *Cox* regression analysis results. GO: Gene Ontology; KEGG: Kyoto Encyclopedia of Genes and Genomes pathway.

### 2.2 *Cox*分析及风险模型构建

对52个与预后显著相关的ARGs进行单因素*Cox*分析，得到7个与预后显著相关的自噬基因*ADAM12*、*CAMP*、*DKK1*、*FAM83A*、*GJB2*、STRIP2和TFAP2A(*P* < 0.05，图 2C)。对上述7个ARGs进行*LASSO*回归分析识别更稳定的基因，虚线标注处为logλ最小值，该处对应的基因为最佳建模基因，LASSO回归系数均不为0，如图 2D所示，虚线右侧为筛选出可作为最佳建模基因的5个稳定基因。经进一步多因素*Cox*回归分析，我们最终得到5个与肺腺癌预后显著相关的自噬基因，构成了肺腺癌预后风险评分模型，分别是ADAM12(ADAM metallopeptidase domain 12, Coef=0.042)、CAMP(Cathelicidin Antimicrobial Peptide, Coef=-0.081)、DKK1(Dickkopf WNT signaling pathway inhibitor 1, Coef=0.036)、STRIP2(striatin interacting protein 2, Coef=0.027)和TFAP2A(transcription factor AP-2 Alpha, Coef=0.039)(图 2E)。其中CAMP的风险比(hazar ratio, HR) < 1，提示低表达与高风险有关; ADAM12、DKK1、STRIP2和TFAP2A的HR > 1，提示高表达与高风险有关，5个基因的生存分析如图 3A-图 3E所示。根据5个ARGs的风险系数和mRNA表达量计算每个样本的风险评分，风险评分(RiskScore)计算公式为：(0.042* ADAM12表达量)+(-0.081* CAMP表达量)+(0.036* DKK1表达量)+(0.027* STRIP2表达量)+(0.039* TFAP2A表达量)。

**图 3:**
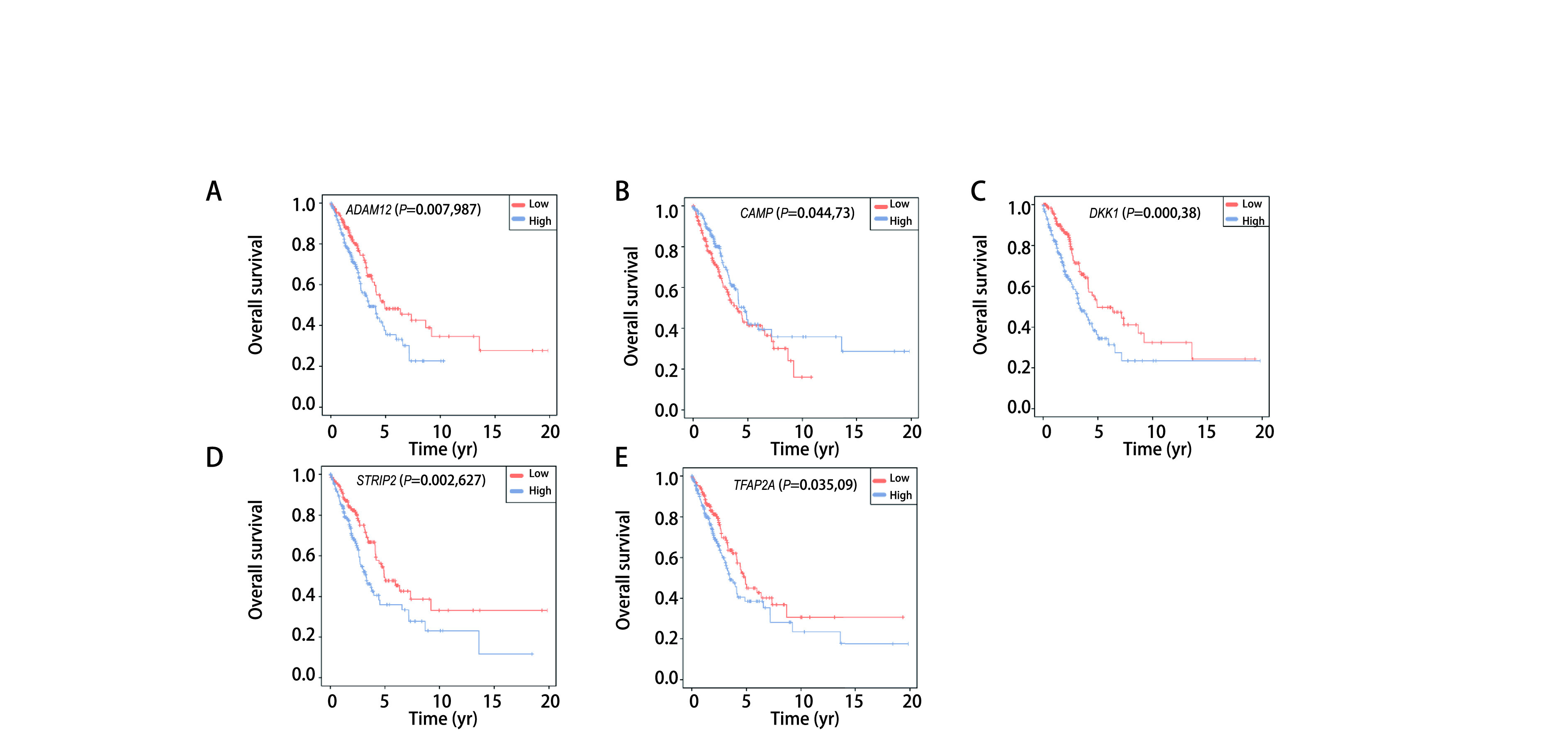
5个建模基因的生存分析。A：*ADAM12*的生存曲线; B：*CAMP*的生存曲线; C：*DKK1*的生存曲线; D：*STRIP2*的生存曲线; E：*TFAP2A*的生存曲线。 Survival analysis of 5 signature genes. A: The survival curve of *ADAM12*; B: The survival curve of *CAMP*; C: The survival curve of *DKK1*; D: The survival curve of *STRIP2*; E: The survival curve of *TFAP2A*.

### 2.3 风险模型性能评价

根据5个ARGs的表达量及回归系数计算出每个肺腺癌样本的风险评分，绘制ROC曲线得到风险评分的最佳cut-off值为1.057，以此将患者分为高风险评分组(*N*=154)和低风险评分组(*N*=227)。可视化分析结果显示，红色代表高风险评分组，蓝色代表低风险评分组(图 4A)。高风险评分组患者死亡比例较低风险组更高，说明高风险评分组更易具有不良预后(图 4B)。ADAM12、DKK1、STRIP2和TFAP2A在高风险评分组高表达，提示高表达与高风险呈正相关; CAMP在高风险评分组低表达，提示低表达与高风险呈正相关(图 4C)，与图 3A-图 3E生存分析结果一致。*Kaplan-Meier*生存曲线表明，高风险评分患者OS明显较低风险评分患者低，二者OS有显著差异(*P* < 0.000, 1，图 4D)。时间依赖性ROC曲线结果如图 4E所示，1年时间AUC为0.78，2年AUC为0.71，3年时间AUC为0.67，5年时间AUC为0.62，7年时间AUC为0.65。上述评价结果表明，该风险评分模型对肺腺癌预后预测有较好的敏感性和特异性。

**图 4:**
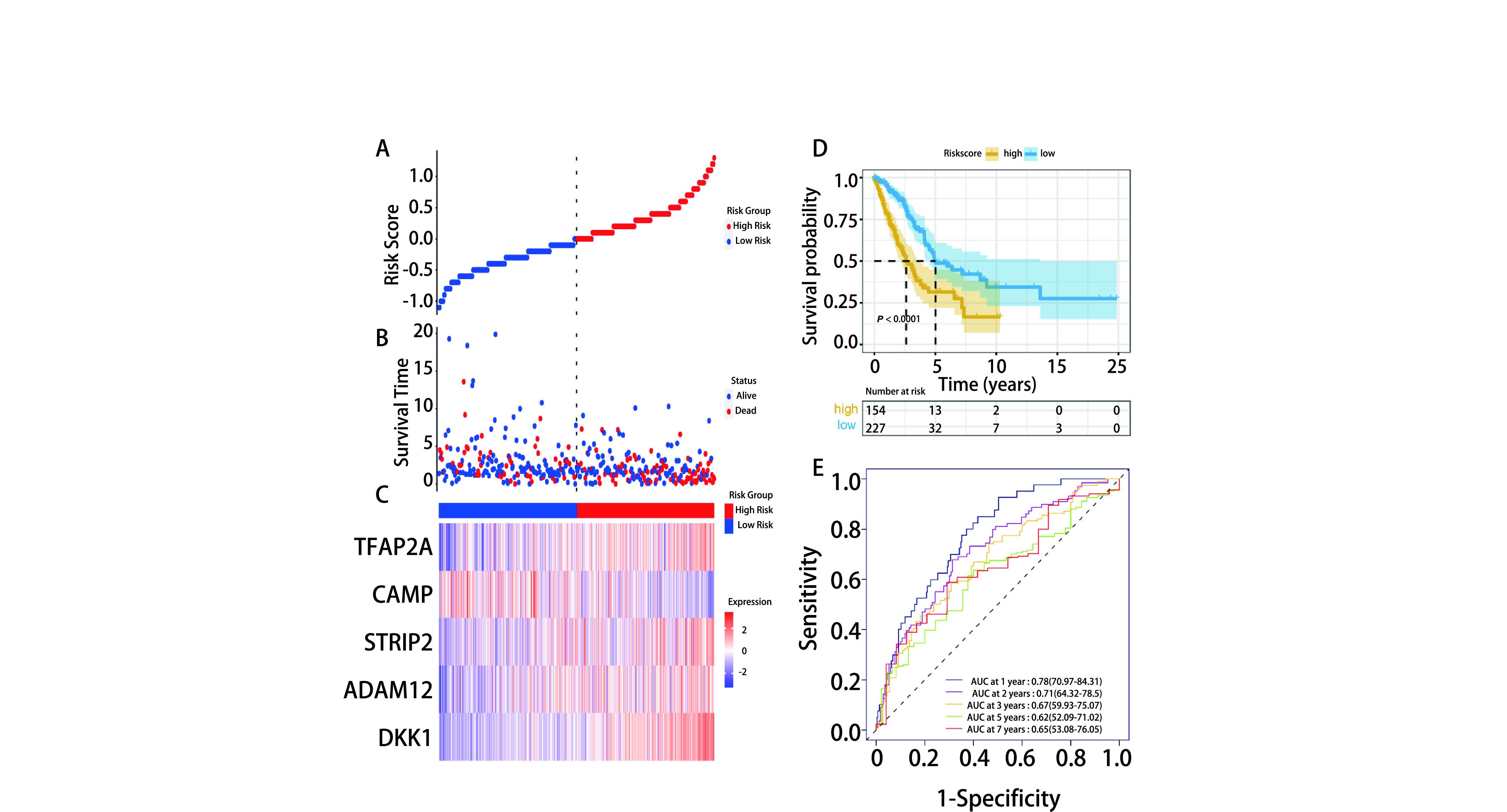
预后风险评分模型的性能评估。A：风险曲线; B：生存状态图; C：建模基因表达热图; D：*Kaplan-Meier*生存曲线; E：时间ROC曲线。 Performance evaluation of prognostic risk score model. A: Risk curve; B: The survival status chart; C: The heatmap of the five signature genes expression profiles; D: *Kaplan-Meier* survival curve; E: Time ROC curve.

### 2.4 风险评分具有独立预后价值

风险评分在单因素和多因素*Cox*回归分析结果均呈现显著差异，说明风险评分具有独立预后价值，可作为LUAD患者的独立预后预测因子。将年龄、性别、肿瘤分期、*EGFR*基因突变、*ALK*基因融合、*KRAS*基因突变和风险评分作为变量纳入单因素和多因素*Cox*回归分析。单因素*Cox*分析显示，风险评分和肿瘤分期是LUAD患者的预后危险因素(*P* < 0.05，图 5A)。再将上述2个危险因素纳入多变量*Cox*分析，结果显示风险评分和肿瘤分期均是LUAD患者的独立预后危险因素(*P* < 0.05，图 5B)。Nomogram图得分可用于推测患者未来1年、3年、5年的生存率(图 5C)。

**图 5:**
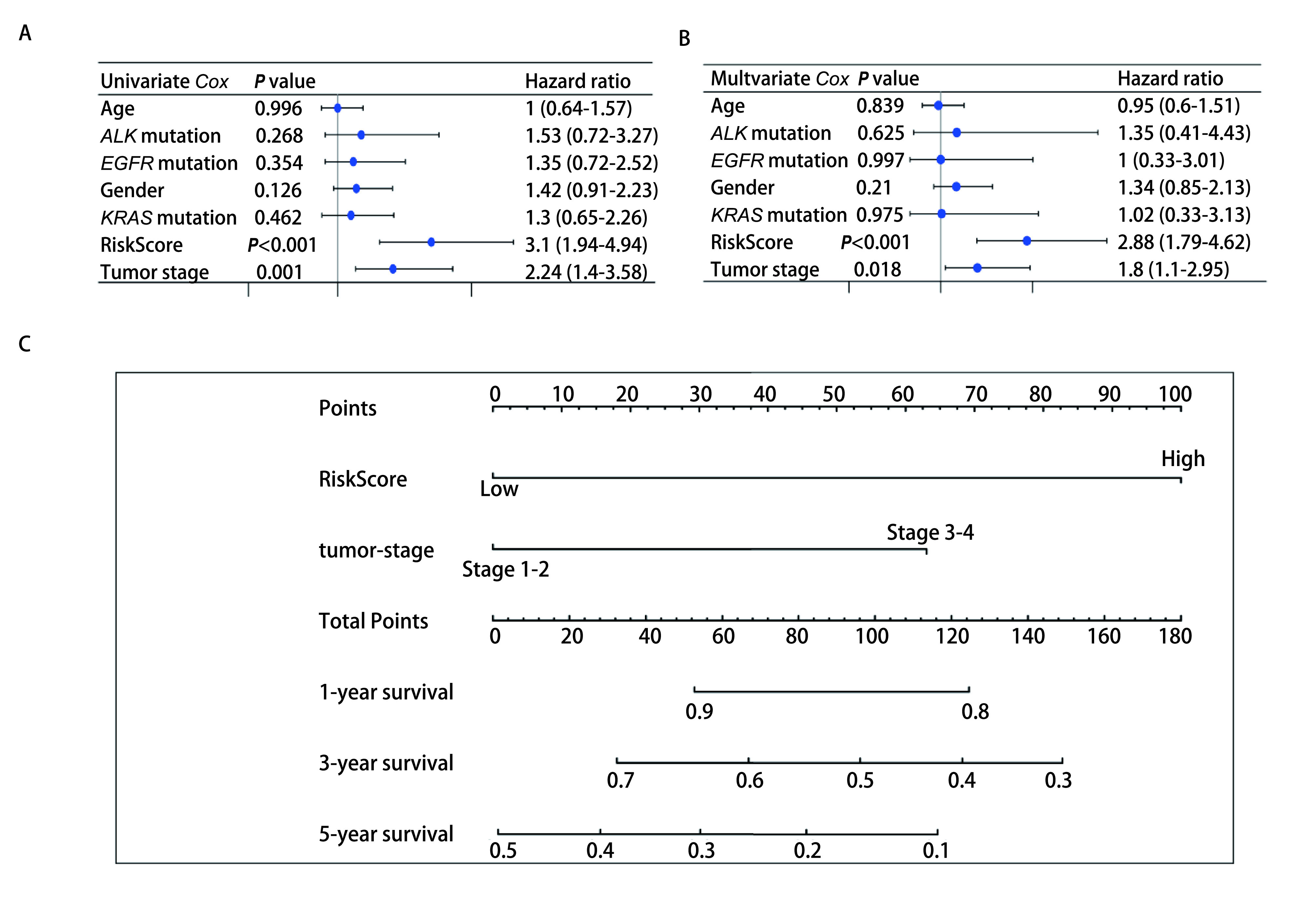
预后风险评分模型的独立预后价值。A：单因素*Cox*独立预后分析; B：多因素*Cox*独立预后分析; C：Nomogram图。 Independent prognostic value of prognostic risk scoring models. A: Univariate *Cox* independent prognostic analysis; B: Multivariate *Cox* independent prognostic analysis; C: Nomogram plot.

### 2.5 风险评分与临床特征相关性

我们利用TCGA-LUAD数据集的数据进一步研究该模型是否对不同临床特征患者的预后也具有预测性能，包括性别、年龄、肿瘤大小、有无淋巴结转移、肿瘤分期、*EGFR*基因突变、*ALK*基因融合、*KRAS*基因突变和生存状态。分析结果：T3期-T4期患者较T1-T2期患者的风险评分高且两组之间存在显著差异(*P* < 0.05，图 6C); T3期-T4期肿瘤患者较T1期-T2期肿瘤患者的风险评分高且两组之间存在显著差异(*P* < 0.05，图 6B); 死亡患者较生存患者的风险评分高且两组之间存在显著差异(*P* < 0.001，图 6A); 而在不同性别、不同年龄阶段、有无淋巴结转移、有无*EGFR*基因突变、有无*ALK*基因融合，及有无*KRAS*基因突变患者的风险评分未出现显著差异。上述结果说明，风险评分与T分期、肿瘤分期和发生不良预后密切相关。

**图 6:**
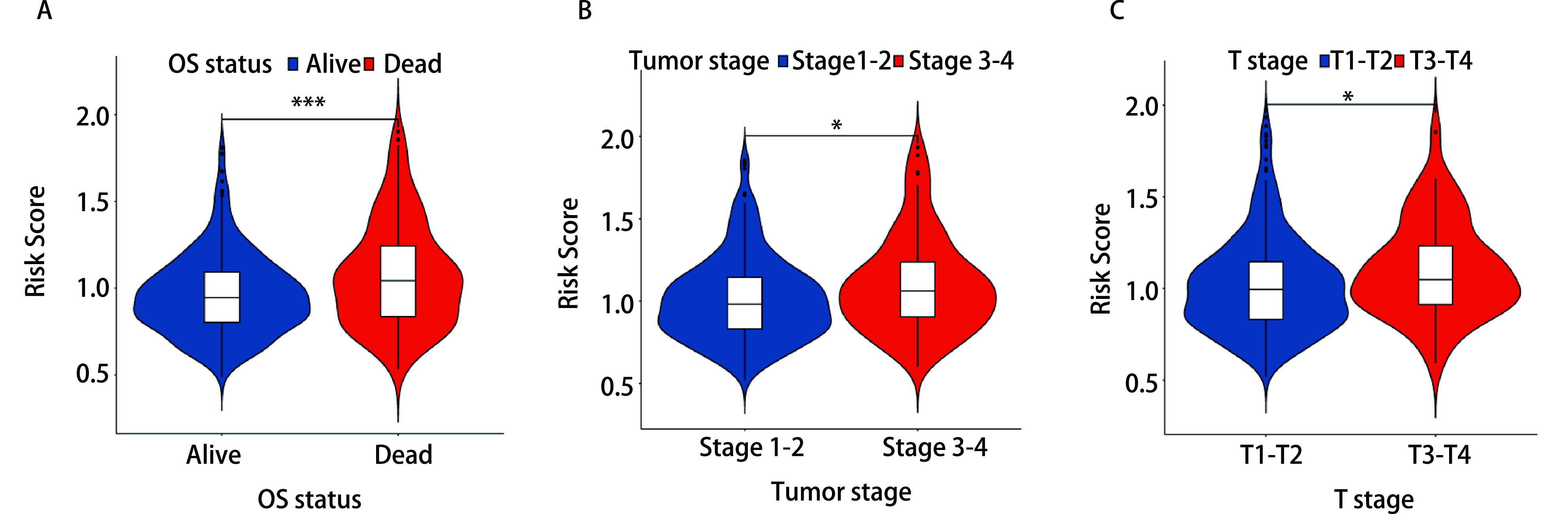
临床相关性分析。A：风险评分与生存状态的临床相关性; B：风险评分与肿瘤分期的临床相关性; C：风险评分与T分期的临床相关性。^*^P < 0.05，^***^P < 0.001。 Clinical characteristic correlation analysis. A: The clinical correlation between risk score and survival status; B: The clinical correlation between risk score and tumor stage; C: The clinical association between risk score and T staging. ^*^P < 0.05, ^***^P < 0.001.

### 2.6 外部数据集验证模型

在GEO数据库中选取GSE31210和GSE72094数据集及作为外部验证集。GSE31210和GSE72094数据集的风险评分cut-off值分别为0.878和1.009，上述两个验证集的*Kaplan-Meier*生存曲线均表明，高风险评分患者OS较低风险评分患者更低，二者OS有显著差异(图 7B，*P*=0.014;图 7D，*P* < 0.000, 1)。两个验证集的1年-5年AUC值在0.61-0.88，说明该模型在外部验证集中仍具有较好的预测性能(图 7A，图 7C)。

**图 7:**
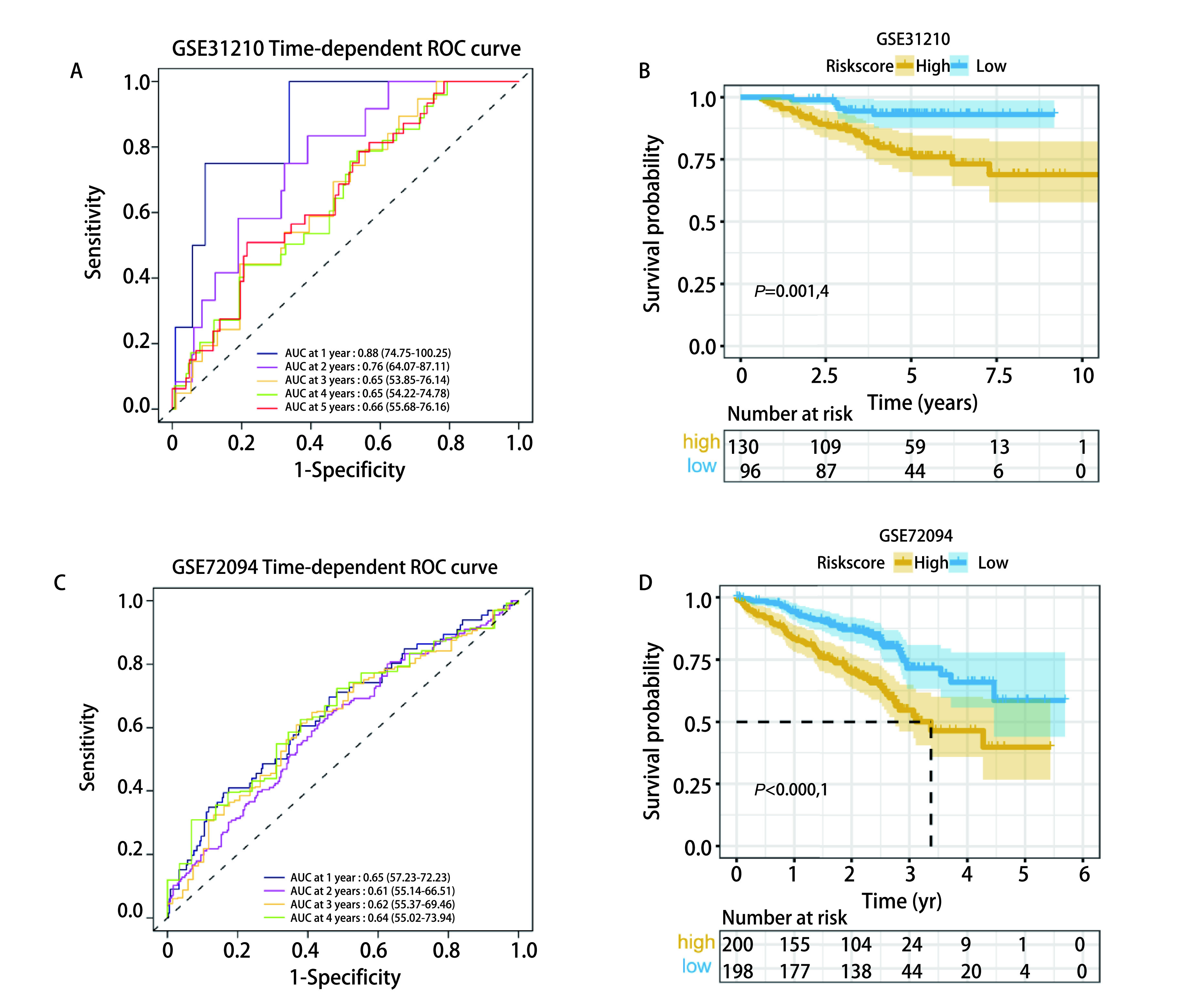
预后风险评分模型在外部验证集中的性能评估。A：GSE31210的时间ROC曲线; B：GSE31210的*Kaplan-Meier*生存曲线; C：GSE72094的时间ROC曲线; D：GSE72094的*Kaplan-Meier*生存曲线。 Performance evaluation of prognostic risk score model in external validation sets. A: Time ROC curve of GSE31210; B: *Kaplan-Meier* survival curve of GSE31210; C: The time ROC curve of GSE72094; D: *Kaplan-Meier* survival curve of GSE72094.

## 3 讨论

研究^[10]^表明自噬参与肺腺癌的发生发展，可以满足肿瘤细胞高代谢的需求，在肿瘤的生长和侵袭中发挥重要作用。自噬是肺腺癌治疗过程中耐药的关键调控因子，抑制自噬可激活*EGFR*突变从而提高Afatinib在肺腺癌中的抗肿瘤活性^[11]^。抑制自噬还可以提高Shh抑制剂vismodegib对LUAD的疗效^[12]^。

本研究通过GeneCard数据库收集ARGs，利用来自TCGA的肺腺癌RNA-seq数据和生存信息，筛选出52个有预后价值的ARGs，经GO和KEGG富集分析提示这些基因主要富集在调控细胞周期、参与自噬、参与HIF-1信号通路和p53信号通路等功能。通过单因素*Cox*回归分析、*LASSO*回归和多因素*Cox*回归分析筛选出5个关键ARGs(ADAM12、*CAMP*、DKK1、STRIP2和TFAP2A)，构建了肺腺癌预后风险评分模型。ADAM12的分泌形式在肺癌中高表达，可促进肿瘤细胞的增殖、迁移和侵袭^[13]^。沉默ADAM12可通过激活人绒毛膜癌JEG-3细胞自噬促进细胞凋亡。抑制ADAM12可降低小细胞肺癌细胞增殖，促进细胞凋亡^[14]^。CAMP是体内的一种宿主免疫肽，具有抗肿瘤作用。CAMP的C端肽LL-37是体内唯一的抗菌肽，在细胞趋化、血管生成、免疫介质诱导和炎症反应调节中发挥重要作用^[15]^。有研究^[16, 17]^发现，LL-37在正常结肠黏膜中表达强烈，在结肠癌组织中表达下调，LL-37可诱导结肠癌细胞凋亡和自噬性死亡，具有独特的抗肿瘤发生作用。DKK1是Wnt信号的负调控因子，是β-catenin/TCF通路的一个靶点，DDK1可通过抑制Wnt-CTNNB1信号通路诱导自噬^[18, 19]^。STRIP2可调节多种肿瘤细胞的生长和迁移。STRIP2在肺腺癌中高表达，通过调控AKT/mTOR通路和上皮-间质转化促进肺肿瘤的增殖和侵袭^[20]^。TFAP2A在多种癌症中均异常表达，例如，TFAP2A在人鼻咽癌中过表达，通过调节HIF-1α介导的VEGF/PEDF信号通路促进肿瘤的发生^[21]^。既往研究^[22]^发现TFAP2A可诱导KRT16过表达，通过EMT促进肺腺癌的发生发展。

上述5个风险ARGs的生存分析和风险评分分布图提示，*CAMP*基因低表达和*ADAM12*、*DKK1*、*STRIP2*、*TFAP2A*基因高表达患者的风险评分高，更易发生预后不良(*P* < 0.05)。通过绘制风险评分分布、*Kaplan-Meier*生存曲线证明高风险评分较低风险评分患者的预后更差，1年、2年、3年、5年和7年时间AUC证明模型对肺腺癌预后预测有较好的敏感性和特异性，并在外部数据集GSE31210和GSE72094得到验证，证明模型的预测性能具有一定的准确性。同时，我们还对风险评分和其他临床预测指标进行了单因素和多因素*Cox*回归分析，证明了风险评分具有独立预后价值，可作为LUAD患者的独立预后预测因子。TNM分期是国际公认的临床预后预测指标，尽管从单因素和多因素*Cox*分析上看风险评分(*P* < 0.001)较肿瘤分期(*P*=0.018)更有优势，但尚不能说明本模型一定优于TNM分期的预测能力。因为本模型尚处于初步建立阶段，且为回顾性研究，样本量较少，仍需要大规模的前瞻性临床试验数据来验证其预测能力是否较TNM分期更好。待完善基础实验后，未来在临床应用中或可与TNM分期联合应用于肺腺癌患者的预后预测。风险评分与临床特征相关性分析结果提示，风险评分高低与T分期、肿瘤分期和发生不良预后密切相关，但它们之间的因果关系仍需进一步探索。同时，有无*EGFR*、*ALK*、*KRAS*基因突变，在单因素和多因素*Cox*回归分析，以及与风险评分进行相关性分析中均未呈现显著差异，此结果可能与临床中的观察并不吻合。分析其原因，可能为包含基因突变信息的样本量较少所导致。

综上所述，本研究通过*LASSO*和*Cox*回归分析构建了基于自噬相关基因组肺腺癌的预后风险评分模型，该模型的预测性能稳定，具有的独立预后价值和临床相关性，可辅助为LUAD患者的个体化诊疗提供参考。与同类研究相比，本研究的LUAD预后风险评分模型存在如下特点：首先，许多同类研究是以免疫为背景，构建免疫相关基因(immune related genes, IRGs)风险模型以预测LUAD患者的预后^[23, 24]^。然而，很少有预测模型以ARGs为基础构建预后预测模型。许多ARGs可调控肿瘤的发生发展，肿瘤组织中ARGs的表达情况在预测生存预后方面具有很大的前景，这些ARGs可作为新的分子靶点。因此，本研究以自噬为背景，筛选与预后显著相关的ARGs，并构建了包含多个ARGs的风险评分模型来预测LUAD患者的生存预后。同时，用于建模的风险基因也可作为LUAD基础研究和潜在的治疗靶点。因此，本研究补充了ARGs风险评分模型在LUAD中的研究空白，以实现对LUAD患者更精准的预后评估，为其个性化治疗提供重要参考。其次，本研究通过2个外部数据集验证所构建的风险模型的可靠性。通过对风险模型的预测效能验证(AUC均值> 0.600)，证明该模型在其他独立数据集中也具有中等程度的预测性能，而上述前人研究中并未在多个数据集中进行验证。遗憾的是，我们的研究仍存在一些局限性。首先，本研究中分析的所有数据均来自公共数据库，所构建的风险模型仍需大规模的临床试验以评估其预测效能; 其次，本研究用于建模的风险基因尚缺少体内、体外实验进一步验证。
